# Effect of Social Support on the Psychological Adjustment of Chinese Left-Behind Rural Children: A Moderated Mediation Model

**DOI:** 10.3389/fpsyg.2020.604397

**Published:** 2021-01-26

**Authors:** Zhiyu Fan, Xinghua Fan

**Affiliations:** ^1^School of Educational Science, Hunan Normal University, Changsha, China; ^2^School of Psychology, Northeast Normal University, Changchun, China; ^3^Cognition and Human Behavior Key Laboratory of Hunan Province, Hunan Normal University, Changsha, China

**Keywords:** left-behind rural children, social support, psychological adjustment, resilience, basic psychological need satisfaction

## Abstract

There are tens of millions of children left behind by one or both rural-to-urban migrant parents (left-behind children, LBC) in rural China. Compared to non-left-behind children (NLBC), LBC are disadvantaged in psychological adjustment. Research has shown social support can help LBC grow up healthily, but the plausible mechanisms linking support to adjustment remain unclear. The present study investigated the mediating role of basic psychological need satisfaction (BPNS) in the above relationship, as well as whether the predictive pathways of support on adjustment and BPNS were moderated by resilience in a sample of 692 LBC and 264 NLBC. Structural equation modeling indicated social support positively predicted LBC’s psychological adjustment, which was completely mediated by BPNS. In addition, the mediating effect was weaker for LBC with higher levels of resilience, which indicated resilience was a necessary asset for LBC’s growth amid the adversity of low social support.

## Introduction

The advancement of industrialization and urbanization in China has caused the migration of rural laborers to the city to seek jobs or start businesses. In 2017, there were 286.52 million migrant workers from rural areas in China (National Bureau of Statistics of China, 2018). Due to poor economic conditions, most of their children were left in rural hometowns under the supervision of one of their parents, grandparents, relatives, or neighbors more than three consecutive months. These children are called left-behind rural children [i.e., left-behind children (LBC)] (The Ministry of Education of the People’s Republic of China, 2016). The Ministry of Education of the People’s Republic of China (2016) reported that there were 20.19 million LBC in the stage of compulsory education in 2015, accounting for 14.42% of children nationwide.

Left-behind children show poor psychological adjustment due to long-term parent–child separation, which has recently attracted increasing concern. Psychological adjustment refers to the phenomenon that an individual will maintain good mental health despite changes in his or her environment (Yuan et al., 2010). It is often measured using different indexes such as depression, happiness, and other emotional indicators (Moztarzadeh and O’Rourke, 2015; Su et al., 2017). Previous studies have shown that compared with those children living in non-migrant families [i.e., non-left-behind children (NLBC)], LBC exhibit more negative emotions such as loneliness and depression, but less positive emotions such as self-esteem and happiness (Liu et al., 2015; Dai and Chu, 2016). Hence, LBC are generally inferior to NLBC in terms of psychological development. Based on the above results, loneliness, depression, self-esteem, and happiness were used as indicators of psychological adjustment in this study.

According to the theory of developmental assets (TDA), LBC’s poor psychological adjustment may be related to their insufficient “assets.” The “assets” in this study refer to the factors that effectively promote the growth of adolescents, including 20 external assets such as family support and 20 internal assets such as resistance skills (Leffert et al., 1998), each of which can positively affect an individual’s adjustment. The individual’s developmental needs and psychological adjustment will be impeded if such assets are absent. Leffert et al. (1998) further emphasized that the assets do not work in isolation, but they may function as a “precursor” to other assets or increase/decrease the efficiency of other assets.

The long-term parent–child separation may result in insufficient care, lack of support assets (Zhao et al., 2018), some unsatisfied basic psychological needs (BPNs) in LBC, and consequently low psychological adjustment. However, some LBC with poor assets show surprisingly good adjustment in daily life. In this case, does social support have a vital influence on the psychological adjustment of LBC? If so, does it function through the mediating effect of basic psychological need satisfaction (BPNS)? In addition, does resilience buffer the adverse effects of deficient support assets on BPNS and psychological adjustment? TDA provides a theoretical basis for this study in exploring these research questions.

### Relation Between Social Support and Psychological Adjustment

Sarason et al. (1983) defined social support as “the existence or availability of people on whom we can rely, people who let us know that they care about, value, and love us.” The main effect model of social support suggests that social support will improve individuals’ psychological health regardless of their current level of stress and support (Cohen and Wills, 1985).

There are four sources of social support for LBC in their daily life. First, migrant father and/or mother communicates regularly with LBC via phone or social media (e.g., WeChat) to learn about their children’s development situation and provide guidance. Meanwhile, the actual guardians may fulfill the parenting and supervision obligations. Second, LBC usually have their own circle of friends to help them when they feel sad (Fan et al., 2018). Third, teachers in the schools typically care for LBC in terms of life, emotions, and academics (Wang et al., 2016). Fourth, the direct relatives such as grandparents or uncles of LBC and social workers may be willing to provide help in their life and studies (Zhang, 2014). Totally, family members, friends, teachers, and significant others all can give them necessary help if LBC need.

Previous studies have demonstrated that the parent–child cohesion and friend companionship negatively predict depression and loneliness of LBC but positively predict their life satisfaction (Zhao et al., 2013, 2015). In addition, the teacher–student relationship has a positive effect on LBC’s self-esteem and a negative impact on LBC’s depression (Liu et al., 2015). Moreover, support from significant people is also crucial to LBC’s emotional and social development (Wang and Mao, 2015). Finally, social support is negatively associated with the mental health problems of LBC (Ye et al., 2017). In general, all the four sources of social support have an important impact on LBC’s psychological adjustment. However, these discrete results make it difficult to reveal the overall relation between social support and psychological adjustment. The ecological systems theory proposed that the relationship between individual and certain microsystem (e.g., family) may influence the relationships between individual and other microsystems (e.g., school) (Bronfenbrenner, 1979). As predicted by this theory, parent–child separation affects not only family support but also other sources of social support, which can be proved through the strong positive correlations (*r* = 0.41∼0.83) between them in both LBC and NLBC (Zhao et al., 2008; Miao et al., 2020). Therefore, this study aimed to use the covariance structural equation model (SEM) to show the full picture of the social support of children in left-behind situation. Based on above results, hypothesis 1 was proposed: social support positively affect LBC’s psychological adjustment.

### Mediating Effect of BPNS

Self-determination theory (SDT) proposes that individuals have three BPNs, namely, need for autonomy (i.e., the desire to dominate their own behaviors without coercion from others), need for relatedness (i.e., the desire to build and maintain intimacy with others), and need for competence (i.e., the desire to accomplish something important) (Ryan and Deci, 2001). These three BPNs are important to individuals’ development, and satisfying these needs contributes to their well-being. Furthermore, the process of need satisfaction does not occur automatically but through interactions between the individuals with their environment (Deci and Ryan, 2012). Indeed, the perception of respect, care, help, and love that children gain through interactions with others not only satisfies their BPNs but also further promotes their psychological adjustment.

As an analogy, Chen et al. (2019) found that BPNS mediates the relationship between perceived need support from physical education teachers and adolescents’ health-related outcomes. In addition, Shao et al. (2018) demonstrated that the parent–child cohesion has a negative impact on LBC’s depression and loneliness through the partial mediation of BPNS. These results indicate that the support of teachers and family members has an effect on adolescents’ psychological adjustment. In daily life, LBC may also interact with their friends frequently and if necessary seek support from significant others such as relatives and caring people, apart from interaction with their schoolteachers and migrant parents. According to the main effect model of social support, the support from different sources has a positive effect on children’s psychological adjustment. Furthermore, in SDT, all the above effects should be achieved through BPNS. Therefore, different sources of social support may positively affect LBC’s psychological adjustment through the mediation of BPNS, which is the hypothesis 2 in this study.

### Moderating Effect of Resilience

“Resilience” is a very broad term that encompasses different essentials in different domains (Masten, 2014). In developmental studies, Masten (2014) defined resilience as “the capacity of a dynamic system to adapt successfully to disturbances that threaten system function, viability, or development” (p. 10). Resilience is interiorly similar to “resistance skills,” a social competency that resists dangerous situations in view of TDA and belongs to internal assets (Leffert et al., 1998, p. 212). Therefore, both resilience (internal asset) and social support (external asset) are protective factors for the development of an adolescent. The protective–protective model (PPM) suggests that two interaction modes exist when several protective factors jointly act on the development outcome, that is, one protective factor may enhance or weaken the impact of another factor on the outcome variables. The former is called promotion hypothesis, and the latter is called exclusion hypothesis (Bao et al., 2013). Wang et al. (2018) found that resilience strengthened the influence of social support on the mental health of junior high school students, which supported the promotion hypothesis. Other studies have suggested that resilience weakened the predictive effect of social support on LBC’s loneliness (Ai and Hu, 2016) and migrant children’s depression and loneliness (Zhao et al., 2014) and they supported the exclusion hypothesis. However, the insignificant moderating effect of psychological resilience on the relation between social support and subjective well-being of the “Ant Tribe” group could not be explained by any hypothesis (Kou, 2012). Hence, the moderating mode of resilience on the direct relation between social support and psychological adjustment remains unclear.

Basic psychological need satisfaction is not only the antecedent variable of psychological adjustment but also the outcome variable of environmental factors. Limited evidence is available on the moderating role of resilience in the relation between social support and BPNS. However, previous studies have demonstrated that individuals with high resilience have additional positive qualities, such as self-confidence and decisiveness (Sivadasan and Narayanan, 2016), good emotional stability and high agreeableness (Van den Akker et al., 2013), strong cognitive ability in interpersonal relationships (Xi et al., 2011c), and implicit tendency to consider themselves capable (Xi et al., 2011a). These traits are conducive to individuals with high-level resilience to obtain the social support assets, achieve behavioral autonomy, establish and maintain intimate relationships with others, and realize the development goals of their ability. As a result, their BPNs are fully satisfied. Under this circumstance, the impact of inadequate external social support from family members, friends, teachers, and significant others on their BPNS should be limited due to their abundant internal assets. By contrast, low-resilience individuals have poor psychological assets, and their cognitive responses to adversity are slow so that they are more likely to assess the influence of adversity to be more persistent (Xi et al., 2011b). Such adversities may easily fixate their attention and become difficult to overcome, and external support may be crucial in their BPNS in this case. Accordingly, resilience may likely reduce the impact of social support on BPNS.

In the same sample, the two moderating effects of resilience on the impact of social support on psychological adjustment and BPNS are simultaneously carried out. However, to the best of our knowledge, limited studies have examined these effects. As far as LBC are concerned, they have been living in a situation with absent support assets for a long time. For one thing, the difficulty in meeting some of their BPNs affects their subsequent adjustment. And, for another, the effect of individual differences in resilience on adjustment has emerged, that is, compared to those with lower resilience, children with higher resilience show less loneliness and better mental health (Ai and Hu, 2016; Luo et al., 2016). It is to be determined whether the moderating effects of resilience on the direct and indirect relationships between social support and psychological adjustment result in LBC’s differentiated development or not. Bronfenbrenner (1979) emphasized that the interaction between environmental factors and individual factors has a great impact on individual’s development, and this interaction may have different strengths and forms in different scenarios. To be specific, LBC with different individual characteristics (resilience) may treat environmental factors (social support) in different ways. Through daily interviews with LBC in dilemma, Fan (2012) found that an old Chinese proverb very aptly summed up the plight of these children: “Jin Shang Tian Hua Yi, Xue Zhong Song Tan Nan,” which means “icing on the cake” is ordinary but “offering fuel in the snowy weather” is scarce. Specifically, for those resilient (usually well-adjusted) LBC, external support only makes their good adjustment even better, so they do not pay much attention to it. However, for those with poor resilience (usually ill-adjusted), they are more eager for external support and cherish this hard-won support more, so the impact of social support on their BPNS and adjustment is likely to be greater. Based on the above viewpoints, hypothesis 3 is proposed: resilience diminish the direct and indirect relations between social support and psychological adjustment in LBC.

### Current Study

Studies have proven the significant impacts of family socioeconomic status (SES) and gender on the psychological adjustment of children (Bradley and Corwyn, 2002; Zhou and Liu, 2015). However, whether school age has significant impact on LBC’s adjustment (e.g., loneliness and depression, Fan and Wu, 2020) remains unclear. Therefore, the current study uses SES, gender, and school age as the candidates of covariables (to be tested), to control their effects on the psychological adjustment of LBC.

In addition, Chang and Zhang (2013) summarized that development assets have different influence patterns and degrees on adolescents’ development due to their different developmental context and history. Similarly, Masten (1999) argued that how resilience works for one group of children may not work for another group of children in a different context. Therefore, this study set NLBC as control group to detect the possible specificity of children in left-behind context and then propose more targeted interventions.

**FIGURE 1:**
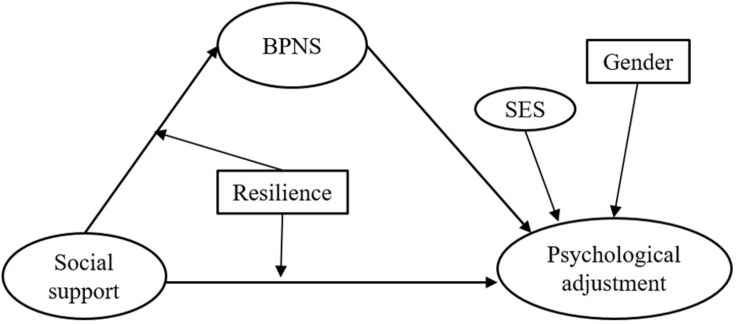
Hypothesized moderated mediation model.

In summary, after controlling for the effects of SES and gender, the current study investigates the following issues: (1) the impact of social support on LBC’s psychological adjustment, (2) the mediating role of BPNS in the relation between social support and psychological adjustment, and (3) the moderating role of resilience in the direct and indirect relationships between social support and psychological adjustment. Figure 1 shows the overall study model.

## Materials and Methods

### Participants

The participants of this study were Chinese rural children (956) aged 8 to 17 years old (*M*_*age*_ = 11.99, SD_*age*_ = 1.73) and enrolled in grades 4 to 9 from 98 rural villages in Hunan Province, China. The criteria of inclusion in this study were (a) normal intelligence (according to the judgment of actual guardian such as grandmother), (b) effective questionnaire response, and (c) from an intact family. According to the migrant situation of their parents, the children are divided into three categories of single-parent migrant [single-LBC (SLBC)], double-parent migrant [double-LBC (DLBC)], and non-migrant parents [non-LBC (NLBC)] (Fan et al., 2018). Table 1 shows their gender and school age distributions. The educational levels of their fathers and mothers were as follows: 18.2% and 31.2% were primary school graduates or below (drop out), 50.0% and 43.0% were junior high school graduates, 16.4% and 11.1% were high school or technical school graduates, 3.3% and 2.8% were junior college graduates or above, and the rest 10.7% and 10.5% were unknown.

**TABLE 1:** Gender and school age distributions of the participants (*n*, %).

	**SLBC**	**DLBC**	**NLBC**	**Total**
Male	155 (47.8%)	171 (46.5%)	108 (40.9%)	434 (45.4%)
Female	169 (52.2%)	197 (53.5%)	156 (59.1%)	522 (54.6%)
Primary school	165 (50.9%)	194 (52.7%)	138 (52.3%)	497 (52.0%)
Junior high school	159 (49.1%)	174 (47.3%)	126 (47.7%)	459 (48.0%)
Total	324 (33.9%)	368 (38.5%)	264 (27.6%)	956

### Measures

#### Social Support

Social support was measured with a 16-item Chinese revised version (Su et al., 2017) of the Multidimensional Scale of Perceived Social Support (Zimet et al., 1988). This scale consists of four dimensions (i.e., support from family, friends, teachers, and significant others) with four items in each dimension (e.g., “My family really tries to help me.”). The participants rated each item on a five-point Likert scale from 1 (*completely inconsistent*) to 5 (*completely consistent*). The average score of each dimension (α ranging from 0.78 to 0.91) was calculated to create an indicator for the latent variable “social support,” with higher values representing higher levels of support.

#### Basic Psychological Need Satisfaction (BPNS)

Basic psychological need satisfaction was measured using a simplified version of the Need Satisfaction Scale (Sheldon et al., 2001). This scale consists of nine items in three dimensions (i.e., need satisfaction for autonomy, competence, and relatedness) with three items in each dimension (e.g., “During this event I felt that my choices were based on my true interests and values.”). The responses were provided on a seven-point Likert scale ranging from 1 (*totally disagree*) to 7 (*totally agree*). The mean score of each dimension (α ranging from 0.68 to 0.87) was calculated to create an indicator for the latent variable “BPNS” with higher values indicating higher level of need satisfaction.

#### Resilience

Block and Kremen’s (1996) 14-item resilience scale was used to measure the children’s resilience (e.g., “I quickly get over and recover from being startled.”). The responses ranged from 1 (*does not apply at all*) to 4 (*applies very strongly*). The mean scores were calculated (α = 0.84) with higher values indicating higher level of resilience.

#### Depression

The revised Chinese version (Wang et al., 1999) of Radloff’s Center for Epidemiologic Studies Depression Scale was used to assess depression symptoms in 20 items during the past week (e.g., “I was bothered by things that usually don’t bother me”). The responses ranged from 1 (*occasionally or never*) to 4 (*most of time or continuous*). The average scores were calculated after the answers of affirmative statements were reversed (α = 0.76) with higher scores representing higher level of depressive mood.

#### Loneliness

Loneliness was obtained using the 21-item adolescents’ loneliness scale (e.g., “It’s easy for me to make new friend”) (Zou, 2003), which consists of four dimensions (i.e., social ability perception, peer relationship evaluation, pure loneliness, and degree of unmet important relationships). The participants rated each item on a four-point Likert scale ranging from 1 (*not conform at all*) to 4 (*completely conform*). The mean scores (α = 0.91) were calculated after the affirmative expressions were reversed with higher scores representing a stronger sense of loneliness.

#### Self-Esteem

Self-esteem was measured with the nine-item Chinese revised version of Rosenberg’s Self-Esteem Scale (e.g., “On the whole, I am satisfied with myself.”) (Wang et al., 1999; Ou and Fan, 2018). The responses ranged from 1 (*completely agree*) to 4 (*completely disagree*). The mean scores were calculated (α = 0.69) after the positive statements were reversed with higher scores representing higher level of self-esteem.

#### Happiness

According to Shen (2009), happiness was measured with the following item: “Overall, do you feel that you have lived a happy life in the past year?” The responses ranged from 0 (*completely unhappy*) to 10 (*happy everyday*) with higher values indicating higher level of happiness.

#### Socioeconomic Status (SES)

Socioeconomic status was calculated from parents’ educational level, parents’ occupation, and family income following the five steps specified in PISA 2003 Technical Report (OECD, 2005) and Fang et al. (2008). First, all variables were assigned. Parents’ educational level was scored according to the years of schooling, and occupations were scored in the range of 16 to 90 according to the International Socioeconomic Index (Ganzeboom and Treiman, 1996). Given that most children may have limited knowledge about their family income, it was indirectly measured by evaluating family resources, including computers, televisions, and 10 other daily facilities in their homes. Each item counted as 1 with a total score from 0 to 12. Second, the above assigned variables were filtered or converted. The parent with higher (compared to his/her spouse) years of schooling or higher score in occupational classification represented the parents’ educational level and occupation. Third, item response theory was used to estimate the family resources and subsequently obtain the parameter estimation index. Fourth, the missing values in each variable were processed. The samples with two or more missing values were eliminated. In the sample with only one missing value, the two other variables were regressed. The regression value then replaced the missing value. Finally, educational level, occupation, and family resources were converted into standard *Z* scores for the following principal component analysis. SES was then calculated by using the following formula: SES = (β_1_ × *Z* educational level + β_2_ × *Z* occupation + β_3_ × *Z* family resources)/ε_*f*_, where β_1_, β_2_, and β_3_ are factor loadings, and ε_*f*_ is the characteristic root of the first factor. Higher scores indicated higher level of family SES.

### Collection and Analysis of Data

Ninety-eight undergraduates were recruited as research assistants from a university in Hunan Province, China. This study used a snowball sampling scheme (Wen and Lin, 2012) to recruit rural children in the rural villages. During a week of winter vacation in 2014, the research assistants conducted some household surveys in the village where their families were located. The study was approved by the Research Ethical Committee of the university, and written informed consent was obtained from all participants and their caregivers. During the survey, the research assistants read the items for primary school students to help them understand and encourage their responses at any time. The junior high school students finished the questionnaire by themselves after the experimenters introduced the requirements. After completing the survey, participants were rewarded with stationery including notebooks and pens.

SPSS 22.0 and AMOS 22.0 were used for data cleaning and screening, preliminary analyses, and model verification. Data cleaning and screening included missing value, outliers, and normality analysis of the data. A total of 1,012 rural children participated in the study, with 16 (1.6%) of them being excluded because of missing more than one third of questionnaires, and 40 (4.0%) of them were excluded on account of missing one or more of the dependent variables according to Wen and Lin (2012). Multiple imputation method was used to insert missing data in the independent variables (Royston, 2005). There were no multivariate outliers (±3 standard deviations) (Shao et al., 2018). Test of normality (shown in Table 2) demonstrated that variables follow a weak skewed distribution, and bootstrap maximum likelihood method was used for follow-up analyses to correct the bias caused by non-normality (Fang and Huang, 2010). The preliminary analyses included (a) MANOVA test and (b) Pearson correlation analyses for the study variables in LBC and NLBC. Model verification included tests for (a) measurement model among all latent variables, (b) main effect, (c) mediating effect, and (d) moderated mediating effect models for LBC and NLBC. Then, invariant models were used to conduct model comparisons between the two groups. Resilience and all the dimensions of social support were centered and then multiplied to obtain four product items as the interactive indexes of resilience and social support when testing moderating effect.

**TABLE 2:** Scores of the study variables and comparison among the three categories of children (*M* ± SD) and skewness and kurtosis of variables.

**Children type**	***n***	**FaS**	**FrS**	**TS**	**SOS**	**BPNA**	**BPNC**	**BPNR**	**Resilience**	**Depression**	**Loneliness**	**Self-esteem**	**Happiness**
SLBC➀	324	3.46 ± 0.96	3.49 ± 0.91	3.18 ± 0.93	3.54 ± 1.06	4.53 ± 1.24	4.59 ± 1.17	5.00 ± 1.34	2.81 ± 0.49	1.93 ± 0.41	1.95 ± 0.49	3.57 ± 0.57	7.32 ± 2.18
DLBC➁	368	3.41 ± 0.96	3.39 ± 0.97	3.16 ± 0.98	3.40 ± 1.13	4.39 ± 1.22	4.44 ± 1.24	4.80 ± 1.39	2.74 ± 0.49	1.91 ± 0.42	1.94 ± 0.50	3.59 ± 0.61	7.00 ± 2.41
NLBC➂	264	3.61 ± 1.00	3.56 ± 0.95	3.22 ± 1.00	3.62 ± 1.12	4.68 ± 1.31	4.78 ± 1.14	5.32 ± 1.28	2.95 ± 0.53	1.80 ± 0.40	1.79 ± 0.48	3.73 ± 0.62	7.96 ± 1.88
*F*_(__2_,_953__)_		3.33*	2.67^+^	0.31	3.30*	3.94*	6.10**	11.26***	14.04***	7.74***	9.86***	6.14**	14.82***
*Post hoc*		➁ < ➂	➁ < ➂		➁ < ➂	➁ < ➂	➁ < ➂	➁ < ➂	➁ < ➂	➁ > ➂	➁ > ➂	➁ < ➂	➁ < ➂
Skewness		−0.55	−0.55	−0.10	−0.53	−0.18	−0.16	−0.53	0.11	0.42	0.42	−0.30	−0.75
Kurtosis		−0.30	−0.25	−0.55	−0.55	−0.36	−0.18	−0.24	−0.40	−0.07	0.16	0.23	0.20

Hereinafter the same: ^+^*p* < 0.08, *p < 0.05, **p < 0.01, ***p < 0.001.

*FaS, family support; FrS, friend support; TS, teacher support; SOS, significant other support; BPNA, basic psychological need for autonomy; BPNC, basic psychological need for competence; BPNR, basic psychological need for relatedness.*

*SEs of skewness and kurtosis for all variables were 0.08 and 0.16, respectively.*

## Results

### Preliminary Analysis

#### Comparisons of Study Variables Among the Three Categories of Children

Table 2 presents the scores of study variables in SLBC, DLBC, and NLBC. The children type was set as the grouping variable and all study variables were used as outcome variables for MANOVA analysis. In terms of overall study variables, the main effect of children type was significant (Wilks’ λ = 0.93, *F* = 2.78, *p* < 0.001, η^2^ = 0.034). Further univariate ANOVA analysis showed that SLBC and DLBC had significantly higher depression and loneliness but lower self-esteem and happiness compared to NLBC. In addition, the levels of the social support from family, friends, and significant others, all dimensions of BPNS and resilience of DLBC, were all significantly lower than those of NLBC.

#### Correlation Analysis Among the Variables

Pearson product–moment correlation analysis was conducted among all study variables and covariates (SES, gender, and school age) in SLBC and DLBC. After each correlation coefficient *r* was converted into fisher *Z*_*r*_, *Z* tests were performed to evaluate the difference of *Z*_*r*_ between the two groups. The results demonstrated no significant difference existed in their pairs of *Z*_*r*_. Accordingly, SLBC and DLBC were merged into one group (i.e., LBC) for further analyses. Table 3 lists the Pearson correlations among the variables in LBC and NLBC.

**TABLE 3:** Pearson correlations among all variables of LBC and NLBC.

**Variables**	**1**	**2**	**3**	**4**	**5**	**6**	**7**	**8**	**9**	**10**	**11**	**12**	**13**	**14**	**15**
1 School age		–0.09	–0.07	–0.05	–0.02	–0.08	0.08	0.07	0.00	0.04	0.05	0.02	0.07	–0.02	–0.06
2 Gender	–0.03		–0.04	0.06	–0.01	0.15*	–0.08	–0.06	–0.08	−0.12^+^	–0.02	0.02	0.10	–0.06	0.02
3 SES	–0.07	–0.04		0.00	0.04	0.04	0.09	0.16**	0.13*	0.10	0.20***	0.06	−0.12^+^	0.11^+^	0.12^+^
4 FaS	–0.06	−0.10**	–0.02		0.67***	0.55***	0.64***	0.28***	0.32***	0.39***	0.32***	−0.23***	−0.24***	0.20**	0.19**
5 FrS	0.02	−0.14***	0.01	0.63***		0.46***	0.63***	0.17**	0.25***	0.25***	0.27***	−0.16*	−0.23***	0.13*	0.12*
6 TS	–0.04	–0.02	0.02	0.52**	0.45***		0.41***	0.28***	0.24***	0.31***	0.27***	−0.13*	−0.16**	0.10	0.12
7 SOS	–0.04	−0.15***	–0.01	0.54***	0.63***	0.38***		0.24***	0.25***	0.36***	0.25***	−0.16**	−0.27***	0.18**	0.21**
8 BPNA	0.03	–0.06	0.09*	0.24**	0.27***	0.21***	0.25***		0.67***.	0.69***	0.52***	−0.38***	−0.35***	0.43***	0.28***
9 BPNC	–0.05	–0.06	0.09*	0.31**	0.33***	0.29***	0.31***	0.65***		0.67***	0.56***	−0.34***	−0.43***	0.52***	0.27***
10 BPNR	–0.04	−0.11**	0.12***	0.36**	0.30***	0.25***	0.29***	0.63***	0.63***		0.54***	−0.36***	−0.39***	0.36***	0.33***
11 Resilience	–0.01	–0.05	0.01	0.21***	0.24***	0.16***	0.19***	0.39***	0.43***	0.31***		−0.35***	−0.38***	0.44***	0.24***
12 Depression	–0.01	0.07^+^	−0.07^+^	−0.21***	−0.18***	−0.12**	−0.21***	−0.28***	−0.32***	−0.36***	−0.31***		0.59***	−0.42***	−0.33***
13 Loneliness	0.01	0.14***	−0.15***	−0.19***	−0.21***	−0.14***	−0.20***	−0.23***	−0.34***	−0.39***	−0.34***	0.60***		−0.48***	−0.36***
14 Self-esteem	–0.02	−0.10**	0.12***	0.24***	0.21***	0.22***	0.18***	0.43***	0.50***	0.41***	0.45***	−0.45***	−0.45***		0.19***
15 Happiness	−0.08*	–0.04	0.19***	0.15***	0.10*	0.08*	0.09*	0.29***	0.30***	0.38***	0.17***	−0.34***	−0.40***	0.33***	

^+^*p* < 0.08, *p < 0.05, **p < 0.01, ***p < 0.001.

*Correlations below and above the diagonal denote LBC and NLBC, respectively.*

*Gender code: 0 = female, 1 = male; school age code: 0 = primary school, 1 = junior high school.*

Table 3 shows that the correlational patterns of the study variables of LBC and NLBC are highly consistent. Positive pairwise correlations existed among all the dimensions of social support, all the dimensions of BPNS, and resilience in both groups, and these variables negatively correlated with depression and loneliness but positively correlated with self-esteem and happiness. The majority of these correlations were significant at the level of 0.001. Moreover, both gender and SES were significantly correlated with depression, loneliness, and self-esteem in LBC. However, there was no significant correlation between school age and these three indicators. On the basis of these correlations, SES and gender were set as the covariates for subsequent analyses.

### Model Verification

#### Measurement Model Among All Latent Variables

**FIGURE 2:**
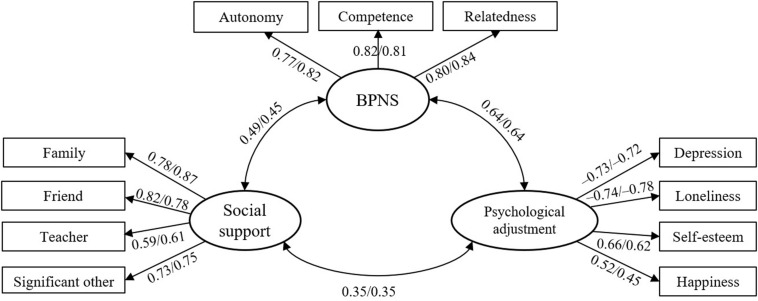
Measurement model among all latent variables. The values before and after the slash “/” denote the path coefficients of LBC and NLBC, respectively, the same below. Model fitting: χ^2^/df = 4.88/2.05 and RMSEA = 0.075/0.063.

As mentioned earlier, the latent variable “social support” included four sources of different support; BPNS was constructed by three kinds of needs satisfaction; and “psychological adjustment” was represented by the four indicators of depression, loneliness, self-esteem, and happiness. On the basis of these constructs, a measurement model including these three latent variables was built. As shown in Figure 2, according to the criterion of goodness-of-indices in AMOS (Wu, 2009), the model fitted the data well for LBC and NLBC [χ^2^/df = 4.88/2.05, normed fit index (NFI) = 0.93/0.93, relative fit index (RFI) = 0.91/0.91, incremental fit index (IFI) = 0.95/0.96, Tucker–Lewis index (TLI) = 0.93/0.95, comparative fit index (CFI) = 0.95/0.96, and root mean square error of approximation (RMSEA) = 0.075/0.063], and all factor loadings were higher than 0.45 (*p* < 0.001).

#### Main Effect of Social Support on Psychological Adjustment

Structural equation model was used to investigate the direct effect of social support on psychological adjustment. The results demonstrated that the overall model fitted the data well for the two groups (χ^2^/df = 2.92/1.55, NFI = 0.95/0.93, RFI = 0.93/0.90, IFI = 0.96/0.97, TLI = 0.95/0.96, CFI = 0.96/0.97, and RMSEA = 0.053/0.046). In addition, social support had a positive impact on psychological adjustment in LBC and NLBC (β = 0.33/0.36, *p* < 0.001), and thus, the higher the social support, the better the psychological adjustment.

#### Mediating Effect of BPNS Between Social Support and Psychological Adjustment

**FIGURE 3:**
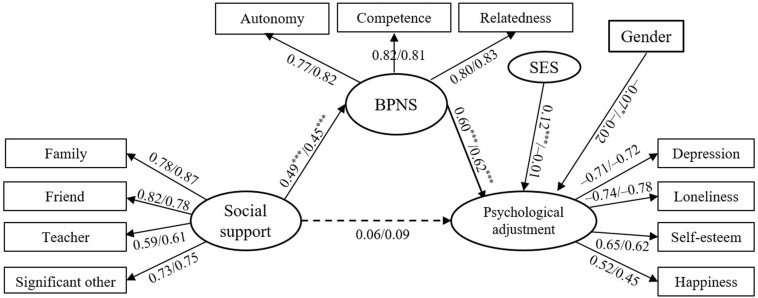
Mediation effect of BPNS on the relation between social support and psychological adjustment in LBC and NLBC. Model fitting: χ^2^/df = 4.14/2.12 and RMSEA = 0.067/0.065. ***p < 0.001.

Figure 3 presents the mediation model linking social support to psychological adjustment with BPNS as the mediator. The results showed that the model of LBC and NLBC both demonstrated a good fit (χ^2^/df = 4.14/2.12, NFI = 0.92/0.90, RFI = 0.89/0.87, IFI = 0.94/0.94, TLI = 0.92/0.93, CFI = 0.94/0.94, and RMSEA = 0.067/0.065). In the two groups, social support had a significant effect on BPNS (β = 0.49/0.45, *p* < 0.001), which in turn had a significant effect on psychological adjustment (β = 0.60/0.62, *p* < 0.001), whereas the direct impact of social support on psychological adjustment (β = 0.06/0.09, *p* > 0.08) was not significant. The bootstrap tests demonstrated that the mediating effect of BPNS was 0.29 and 0.28 in LBC and NLBC, which accounted for 83.6% and 76.6% of the total effect, respectively. Their corresponding 95% confidence intervals (CIs) were (0.21, 0.38) and (0.17, 0.45) at *p* < 0.001, which indicated that BPNS completely mediated the relationship between social support and psychological adjustment in both groups of children.

#### Moderating Effect of Resilience on Social Support→BPNS and Psychological Adjustment

**FIGURE 4:**
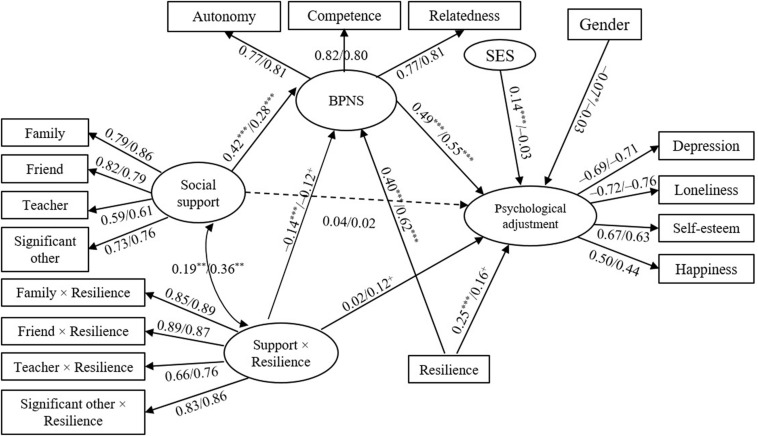
Relation between social support and psychological adjustment in LBC and NLBC: a moderated mediation model. Model fitting: χ^2^/df = 3.62/2.50 and RMSEA = 0.062/0.075. ^+^*p* < 0.08, *p < 0.05, **p < 0.01, ***p < 0.001.

As shown in Figure 4, four paths of resilience and social support × resilience→BPNS and psychological adjustment were added on the basis of the mediation model.

The results showed the model fitting indexes in LBC and NLBC were acceptable (χ^2^/df = 3.62/2.50, NFI = 0.91/0.86, RFI = 0.89/0.85, IFI = 0.93/0.91, TLI = 0.92/0.90, CFI = 0.93/0.91, and RMSEA = 0.062/0.075). The interaction of social support and resilience had a significant effect on BPNS in both groups (β = −0.14/−0.12, *p* < 0.05), whereas the effect of social support × resilience on psychological adjustment was only significant in NLBC (β = 0.17, *p* < 0.05).

Second, simple effect analyses were carried out to detect the interaction effects. LBC and NLBC were ranked from lowest to highest using their *Z* scores in resilience. The children with *Z* score less than/equal to −1 and greater than/equal to 1 were grouped into low- and high-resilience groups, respectively, while the rest belonged to the medium group. We used SEM to verify the mediation model (see Figure 3) in the three groups of LBC and NLBC. Table 4 shows that in the three LBC groups, the direct effects of social support on psychological adjustment were insignificant and the mediating effects were all significant at the 0.01 level, which indicated social support promoted LBC’s psychological adjustment through BPNS regardless of the level of resilience. Notably, the higher mediating effect values of low and medium groups (0.29/0.27) than those of the high group (0.14) demonstrated a generally decreasing trend of mediating ability of BPNS, which reduced significantly in the high-resilience group.

**TABLE 4:** Direct and mediating effects of social support on psychological adjustment in the different resilience levels.

**Resilience**	**LBC**					**NLBC**				
	**Support → Adjustment β (*p*)**	**Support → BPNS β (*p*)**	**Support → BPNS → Adjustment**			**Support → Adjustment β (*p*)**	**Support → BPNS β (*p*)**	**Support → BPNS → Adjustment**		
			**Effect**	**95% CIs**	***p***			**Effect**	**95% CIs**	***p***
Low	−0.16 (0.399)	0.70 (0.000)	0.29	(−0.050, 0.659)	0.061	−0.20 (0.473)	0.68 (0.000)	0.44	(−0.087, 1.518)	0.078
Medium	0.07 (0.264)	0.47 (0.000)	0.27	(0.170, 0.390)	0.001	0.20 (0.025)	0.31 (0.000)	0.18	(0.063, 0.418)	0.007
High	0.08 (0.448)	0.24 (0.022)	0.14	(0.015, 0.341)	0.024	−0.06 (0.703)	0.30 (0.054)	0.12	(−0.022, 0.666)	0.104

*The number of bootstrap samples was 1,500 in all the groups.*

Table 4 also shows that the direct effect of social support on adjustment was only nearly significant in the medium group (β = 0.20, *p* < 0.05) of NLBC. In addition, the main effect of social support on BPNS (0.70 → 0.28 → 0.24) and the mediating effect of BPNS (0.42 → 0.18 → 0.08) declined with the increasing level of resilience, and both became insignificant at a high level of resilience. This finding suggests that the increasing level of resilience reduced the mediating ability of BPNS in turn (complete mediation → partial mediation → no mediation), and social support had no effect on adjustment in the high-resilience group.

Third, a series of consistency tests were conducted to detect the difference of the path coefficients either in the moderated mediation model (see Figure 4) between LBC and NLBC or in the mediation model among the low-/medium-/high-resilience groups of LBC and NLBC (see Figure 3 and Table 4). Invariant models were used to reduce the risk of family-wise errors (Wu, 2009). Results of nested model comparison showed that moderated mediation model and mediation model in medium-resilience group both demonstrated an overall significant change (χ^2^/df_(__9__)_ = 2.60, ΔNFI = 0.003, ΔIFI = 0.003, ΔRFI = 0.000, and ΔTLI = 0.000, *p* < 0.01) when assuming model measurement weights and intercepts to be correct. Further comparison showed that mediation model only demonstrated a significant change in medium-resilience group (χ^2^/df_(__5__)_ = 3.35, ΔNFI = 0.007, ΔIFI = 0.008, ΔRFI = 0.002, and ΔTLI = 0.002, *p* < 0.01). The results of pairwise comparisons showed that the path coefficient of social support → BPNS in LBC was larger than that of NLBC (β = 0.42/0.28, C.R. = 2.21, *p* < 0.05), but this difference was only demonstrated in the medium-resilience groups (β = 0.47/0.31, C.R. = 2.03, *p* < 0.05) with the influence being stronger in LBC than that in the corresponding NLBC group. In addition, the weaker coefficient of resilience → BPNS in LBC than that in NLBC (β = 0.40/0.63, C.R. = 3.18, *p* < 0.001) indicated that resilience had a stronger impact on the BPNS of NLBC.

## Discussion

On the basis of the TDA framework, social support, BPNS, and psychological resilience denoted predictor, mediator, and moderator, respectively. We investigated their joint influences on psychological adjustment in LBC and obtained significant results. The findings of this study will provide a theoretical basis on the mental health intervention of LBC.

### Comparison of LBC and NLBC Scores in the Study Variables

Compared with NLBC, LBC exhibited lower self-esteem and happiness but higher loneliness and depression. Hence, LBC showed poor psychological adjustment, which is consistent with the results in previous studies (Liu et al., 2015; Dai and Chu, 2016; Shao et al., 2018). In addition, LBC exhibited decreased support from family, friends, and significant others and lower resilience, indicating that they had poor external support and internal resistance assets. From the perspective of TDA, the insufficiency of assets may induce poor psychological adjustment (Leffert et al., 1998).

The growth environment in LBC has been changed since their parents’ migration due to employment. LBC may experience not only decreased parental care (Fan et al., 2018) but also high social discrimination (Zhang et al., 2015) and peer bullying (Lu et al., 2017). Some social help activities might also strengthen their feelings of being treated differently. As a result, LBC generally perceived a decreased sense of effective social support. According to the conservation of resources theory (Hobfoll, 2001), the shortage in resource will put a certain pressure on individuals. To alleviate the disadvantages brought by life pressure, LBC must use their limited assets for resistance. However, such a small investment will unlikely make ends meet. The continuous depletion of their anti-adversity ability will likely cause the loss of assets in LBC; hence, their resilience will increasingly decrease (Fan et al., 2017).

Moreover, LBC exhibited significantly lower BPNS than NLBC, which is consistent with the results of Shao et al. (2018). Childhood and adolescence are of great importance for the physical and mental development of individuals, and the children in these two stages are highly dependent on their families and parents. Although LBC have additional opportunities to make seemingly autonomous decisions, such autonomy without parental guidance will likely confuse them and result in the poor satisfaction of the need for autonomy. In addition, the absence of parental care in LBC cannot be compensated by other people (Fan et al., 2018), and the children often face a considerable amount of pressure in life and have lower levels of self-confidence and enterprising spirit than NLBC (Fan et al., 2017). Thus, it is difficult for LBC to fulfill their needs for relatedness and competence.

### Independent Prediction Effect of Social Support on Psychological Adjustment of LBC

In line with our hypothesis 1, social support had a positive impact on LBC’s psychological adjustment. This result is consistent with the proposition of the main effect model in which social support generally has a positive effect on the physical and mental health of individuals. This finding is also congruent with TDA’s assumption of horizontal pile-up effect, which indicates that the additional support assets from different contexts improve the adaptive outcome (Benson et al., 2006). Effective care, help, love, and respect from family members, teachers, friends, and significant others are extremely vital in LBC who are desperate for love at this time. This assistance not only broadens their social space and makes them feel safe, supported, and competent but also protects them from life pressure (Benson et al., 2006) in the pursuit of maintaining good mental health.

### Mediating Effect of BPNS on the Relation Between Social Support and Psychological Adjustment in LBC

In line with our hypothesis 2, BPNS fully mediated the effect of social support on LBC’s psychological adjustment. In other word, the positive effect of social support on psychological adjustment was completely realized through BPNS. This finding can be explained from the viewpoint of SDT. Deci and Ryan (2012) proposed that BPNs are of universal importance in the psychological adjustment of adolescents, and the degree of satisfaction in these needs depends on the adequacy of support assets in their living environment. Specifically, if the support provided by a certain environment cannot meet an individual’s basic needs, they will look for it in another environment. If the other environments cannot provide sufficient support either, they will fall into a dilemma of maladjustment. As far as LBC are concerned, the support they receive from grandparents and migrating parents in family life is extremely limited, and the effective help from friends and teachers in school life is also inadequate. Moreover, in their social life, the substantial assistance from volunteers, such as social workers, is often unsustainable. Therefore, the lack of support assets makes it difficult to satisfy their BPNs and hinders their psychological adjustment.

### Moderating Effect of Resilience on the Relation Between Social Support to LBCs BNPS and Psychological Adjustment

In line with our hypothesis 3, the results showed that the interaction of social support and resilience had a negative effect on BPNS. With the increasing level of resilience, the power of social support to predict BPNS weakens, which changes from “offering fuel in the snowy weather” to “icing on the cake,” thus leading to the reduction in the mediating ability of BPNS. This finding supported the exclusion hypothesis of PPM.

The findings further indicated that resilience reduced the mediation between social support, BPNS, and psychological adjustment. In other words, social support influence on psychological adjustment was less likely to occur through BPNS among LBC who had a higher level of resilience, implying resilience may have a stronger impact on psychological adjustment compared to the mediation pathway. To roughly examine our inference, we added the path “Resilience → Psychological adjustment” to conduct the aforementioned consistency tests in the invariant mediation model among the low-/medium-/high-resilience groups of LBC. Results showed that with the increasing level of resilience (low → medium → high), the mediating ability of BPNS reduced (0.29 → 0.25 → 0.12) and the direct effect of resilience on psychological adjustment increased (0 → 0.20 → 0.30), which is consistent with our inference. Therefore, high level of resilience directly enables LBC to have a high level of adjustment, without relying solely on BPNS to play a mediating role.

The analysis in NLBC (control group) demonstrated that resilience had a simultaneously significant moderating effect on the association of social support with BPNS and psychological adjustment. With the increasing level of resilience, the mediating ability of BPNS reduced (0.44 → 0.18 → 0), and social support no longer had an effect on psychological adjustment in the high-resilience group. The declining trend of this mediating effect in NLBC was nearly consistent with that in LBC (0.29 → 0.27 → 0.14), but the decline rate of the former was clearly greater than the latter. These patterns indicate that the moderating model of resilience is applicable to both groups, but the moderating ability in NLBC is generally better than that in LBC. These conclusions support the TDA’s viewpoint wherein the effects of development assets on adaptive outcomes vary with the context of adolescents (Chang and Zhang, 2013).

Masten (2014) emphasized that if children were exposed to adversity in their early development, especially in the critical period when individual differences in personality and self-regulation were formed, their vulnerability to stress would increase and thus form malignant response patterns to stress and poor self-regulation skills. As far as LBC are concerned, they have been living with the dilemma of insufficient support resources for a long time. Consequently, many of LBC will become sensitive and self-abased, excessively caring about how others judge them with their extreme lack of self-confidence. These personality traits might increase their vulnerability to left-behind dilemmas and hinder the development of resilience. By contrast, NLBC possessed a better growth environment, more adequate support assets, and more developed resilience. Therefore, the moderating effect in NLBC is more evident than that in LBC.

Moreover, it is worth noting that unlike LBC, no increasing trend of effect of resilience on adjustment (0 → 0.16 → 0) was found when conducting the abovementioned consistency tests for NLBC. This suggests that compared with NLBC, although the left-behind dilemma weaken the moderating function of LBC’s resilience, it may make LBC grow from another perspective, that is, make their resilience play a more direct role.

Furthermore, cross-group (LBC vs. NLBC) comparisons showed that the impact of social support on BPNS was significantly greater in LBC than that in NLBC. The reason is that LBC have fewer available support assets and more life stress to cope with (Fan et al., 2017), so they will likely place more importance on their existing support than NLBC. Consequently, changes in support resources will produce improved satisfaction when support increases but demonstrate greater loss when support decreases.

In addition, the smaller influence of resilience on BPNS in LBC than that in NLBC can be elucidated by the different functions of resilience in the two groups. As mentioned above, compared with NLBC, the effect of psychological resilience in LBC is more reflected in directly improving their psychological adjustment, rather than only affecting the paths related to BPNS.

### Significance, Implications, and Limitations

This study explored the influence mechanism of social support on psychological adjustment in LBC and NLBC and tested the viewpoint of the assets model of human positive development. Compared with previous studies that only demonstrated the mediating effect of resilience (Zhao, 2015), the current study found that resilience can alleviate the impact of adverse situations on LBC, which is of more practical significance. On the basis of the results, we now provide a form of intervention on the psychological adjustment of LBC.

First, a sound social support system should be cultivated for LBC. Specifically, we should provide targeted guidance and training in the parent–child communication skills for migrating parents and make efforts to create a fair and harmonious atmosphere in the campus and community (village). For example, a “Community Family Activity Room” in Sichuan Province of China enhanced LBC’s sense of social support by mobilizing the internal strength of rural communities and giving LBC a place to learn, entertain, and exercise (Zhang and Ye, 2010). Meanwhile, we should coordinate and guide all kinds of social care activities that LBC accept willingly.

Second, we should focus on improving the psychological resilience level of LBC. Scales and Leffert (2004) proposed several measures, such as (a) increasing children’s participation in social activities that promote social and psychological development, (b) enhancing their self-efficacy experience, and (c) implementing adult supervision responsibilities to build supportive adult networks that promote the resilience development of children. The abovementioned views can be used and combined with the specific plight of LBC to carry out resilience intervention, which could not only enhance their immunity against life pressure but also alleviate the adverse impact of poor support assets on psychological adjustment. In line with abovementioned assumptive measure (c), Guan and Deng (2019) provided us a good example by implementing a community-based “Children’s Companion Mother Program,” which benefit LBC in many aspects including their resilience.

The major limitations of this study were as follows. First, the data in this study were collected via self-report questionnaires, making it difficult to eliminate the influence of social desirability. In addition, Tian (2006) summarized the Chinese version of Rosenberg’s Self-Esteem Scale’s shortcoming that related to cultural difference, such as item 8 had a different meaning from that of Western version, which lead to the relatively low reliability in self-esteem measure both in the current study (α = 0.69) and other Chinese studies (e.g., Liu et al., 2015, α = 0.75). Subsequent research could be improved by combining self-report questionnaires with other-report methods and adopting more indigenized scales with higher reliability. Second, this investigation used a cross-sectional design, which could only demonstrate the correlation pattern between variables statistically. Follow-up longitudinal studies are needed to verify these findings at the causal level. Third, snowball sampling conducted in the current study is a non-probability sampling, and the data were collected from Hunan province rather than the whole country of China, which may lead to the possible selection bias and limited generalizability of study’s conclusion. Future studies could randomly sample over a larger sampling range. Fourth, some additional variables related to the left-behind phenomenon were not considered. Studies have shown that the age at which children are separated from their parents (Liu et al., 2009), state of communication with migrating parents (Wang et al., 2015), and duration of separation from parents may have a main or interacting effect on their psychological adjustment (Fan et al., 2018). The internal validity of a follow-up study will be improved if the above factors can be controlled.

## Conclusion

First, compared with NLBC, LBC exhibited lower level of social support, BNPS, resilience, and psychological adjustment. Second, the higher the level of social support, the higher the LBC’s psychological adjustment. Third, social support affected LBC’s psychological adjustment completely through BPNS. Fourth, with the increasing level of resilience, the indirect effect of social support on LBC’s psychological adjustment was gradually weakened.

## Data Availability Statement

The raw data supporting the conclusions of this article will be made available by the authors, without undue reservation.

## Ethics Statement

The studies involving human participants were reviewed and approved by Research Ethical Committee of Hunan Normal University. Written informed consent to participate in this study was provided by the participants’ legal guardian/next of kin.

## Author Contributions

XF conceived the research. ZF and XF performed the research, analyzed the data, and wrote the manuscript. Both authors contributed to the article and approved the submitted version.

## Conflict of Interest

The authors declare that the research was conducted in the absence of any commercial or financial relationships that could be construed as a potential conflict of interest.
